# m6A regulator‐mediated RNA methylation modification patterns are involved in immune microenvironment regulation of periodontitis

**DOI:** 10.1111/jcmm.16469

**Published:** 2021-03-16

**Authors:** Xiaoqi Zhang, Shizhen Zhang, Xinyu Yan, Yue Shan, Lu Liu, Jing Zhou, Qianyun Kuang, Minqi Li, Hu Long, Wenli Lai

**Affiliations:** ^1^ Department of Orthodontics West China Hospital of Stomatology State Key Laboratory of Oral Diseases National Clinical Research Center of Oral Diseases Sichuan University Chengdu China; ^2^ Department of Bone Metabolism School and Hospital of Stomatology Shandong University & Shandong Key Laboratory of Oral Tissue Regeneration & Shandong Engineering Laboratory for Dental Materials and Oral Tissue Regeneration Chengdu China

**Keywords:** epigenetics, immune microenvironment, immunity, m6A, periodontitis, RNA modification

## Abstract

The role of epigenetic regulation in immunity is emerging, especially for RNA N6‐methyladenosine (m6A) modification. However, little is known about the role of m6A in the regulation of the immune microenvironment of periodontitis. Thus, we aim to investigate the impact of m6A modification in periodontitis immune microenvironment. The RNA modification patterns mediated by 23 m6A‐regulators were systematically evaluated in 310 periodontitis samples. The impact of m6A modification on immune microenvironment characteristics was explored, including infiltrating immunocytes, immune reaction gene‐sets and HLAs (human leukocyte antigen) gene. m6A phenotype‐related immune genes were also identified. 17 m6A regulators were dysregulated and a 15‐m6A regulator signature can well distinguish periodontitis and control samples. ALKBH5 and FMR1 are closely related to infiltrating monocyte abundance. ELAVL1 and CBLL1 are significant regulators in immune reaction of TNF_Family_Members_Receptors and Cytokine. The expression of HLA‐B and HLA‐DOA is affected by ALKBH5 and LRPPRC. 3 distinct RNA modification patterns mediated by 23 m6A regulators were identified. They differ from immunocyte abundance, immune reaction and HLA gene. 1631 m6A phenotype‐related genes and 70 m6A‐mediated immune genes were identified, and the biological functions of these were explored. Our finding demonstrated the m6A modification plays a crucial role in the diversity and complexity of the immune microenvironment of periodontitis.

## 1 INTRODUCTION

Periodontitis is a chronic inflammation occurring in periodontal supporting tissue, caused by inflammatory responses to bacteria in dental plaque. Without proper management, periodontitis could bring damage to periodontal supporting tissues, leading to the formation of the periodontal pocket, attachment loss, absorption of alveolar bone and eventually tooth loss.^1^ Besides, periodontitis has been proved to be potential pathogenic factors for other systemic diseases such as diabetes, cardiovascular diseases and rheumatoid arthritis. Being the sixth most prevalent health condition, periodontitis is affecting the lives of 10.8% of people worldwide, imposing a massive burden for the economy and society.^2^ The inflammation of periodontal tissue is the result of the interaction between host immune defence reactions and microorganisms, and the pathogenesis of periodontitis is considered to be closely related to immunity.^3^ The innate immunity plays an early defence role, and as the chronic inflammation progresses, the adaptive immune reaction is activated which will mediate the repair and regeneration of damaged periodontal tissues.^4^ Besides, the severity and susceptibility of periodontitis are largely determined by an immune response from the host, especially the degree of immune reactions and the type of activated immune pathways under the stimulation of pathogens.^3^ Thus, understanding immune regulations in periodontitis might be the key to reveal the pathologic mechanisms behind it and might shed some light on uncovering novel immune therapies for periodontitis.

Traditionally, epigenetics included the reversible modification of DNA and protein (histones), which could independently regulate gene expression outside of DNA sequences.^5^ It was not until recently that RNA modification was believed to be the third layer of epigenetics, regulating RNA processing and metabolism.^6^ RNA modification exists in all living forms, and there are more than 150 modifications that have been uncovered, including 5‐methylcytosine (m5C), N6‐methyladenosine (m6A) and N1‐methyladenosine (m1A), among which the most abundant form would be m6A.^7^ m6A modification is a dynamic reversible process in eukaryotic cells regulated by methyltransferases (writers), demethylases (erasers) and binding proteins (readers).^8^ The methylation processes of m6A are regulated by methyltransferases, including METTL14, METTL3 and WTAP, while demethylases control demethylation of m6A consisting of ALKBH5 and FTO. Readers are a group of proteins binding to m6A that could identify the methylation motif of m6A, thus mediating the regulatory functions of m6A, and they have YTHDC families and YTHDF families.^9^

Recent studies have demonstrated that m6A regulation could explain some of the underlying mechanisms of immune regulation. Under the infection of the DNA virus, hnRNPA2B1 recognizes viral DNA and facilitates the m6A modification to trigger innate immune responses.^10^ Besides, m6A was also found to play a vital role in adaptive immunity. YTHDF1 was found to participate in the antigen presentation from dendritic cells to CD8^+^ T cells by enhancing lysosomal cathepsin translation and impairing the cross‐presentation of tumour neoantigens and the cross‐priming of CD8^+^ T cells, which in turn facilitates the immune escape for tumour cells.^11^ Besides, Li et al^12^ discovered that the deletion of METTL3 in T cells could impair the homeostatic differentiation of T cells. Although accumulating evidence has suggested that m6A's regulatory role in immune responses, no current study has focused on what kind of a role m6A might play in the pathogenesis of periodontitis, especially the immune responses against the pathogens in dental plaque. A thorough investigation of the immune alterations between healthy and periodontitis samples as well as among various subtypes of periodontitis, and how the m6A regulators might change in between these alterations, could enhance the understanding of the pathogenic mechanism of periodontitis from a brand‐new aspect.

In this study, we systematically evaluate the modification pattern of m6A regulators in periodontitis. We found that m6A regulators can well distinguish healthy and periodontitis samples. Infiltrating immunocyte abundance and immune reaction gene‐set of periodontitis with significant correlations to m6A regulators were observed, suggesting a tight bond between m6A regulators and immune regulations. We clustered periodontitis samples according to 23 m6A regulators, and 3 distinct m6A modification patterns were identified. Different immune characteristics were observed among these subtypes, and we compared the biological functions in these subtypes. Besides, 1631 m^6^A phenotype‐related genes and their biological functions were investigated. These findings above indicate that m6A modification patterns have significant impacts on the immune microenvironment of periodontitis.

## 2 MATERIALS AND METHODS

### 2.1 Data preprocess

The data used in this study consisted of 310 samples, including 69 healthy samples and 241 periodontitis samples, which came from 120 patients undergoing periodontal surgery. The sample process protocol and RNA extraction protocol were well described in the previous study.^13^ The gene expression was detected by Affymetrix Human Genome U133 Plus 2.0 Array microarray according to the manufacturer's instructions. The data were reserved in the GEO (Gene expression omnibus) database and the serial number is GSE16134 (https://www.ncbi.nlm.nih.gov/geo/query/acc.cgi?acc=gse16134). All data were preprocessed as our previous study did and obtained by R package “GEOquery”.^25^ All CEL files in the series were processed using ‘RMA’ in R using ‘justRMA’ with default parameters. Gene probes were annotated as gene symbols. Probes without matching gene symbols and matching multiple symbols were excluded. Gene expression value of duplicate gene symbol was calculated as the median value. The expression value was preprocessed by the ‘normalizeBetweenArrays’ function in the ‘limma’ package. m6A regulators investigated in this study were referred from the previous studies conclusions.^14^, ^15^, ^16^, ^17^

### 2.2 Alteration analysis of m6A regulator between healthy and periodontitis

The protein‐protein interaction network of 23 m6A regulators was consulted from the STRING database (https://string‐db.org/). The expression relationship among 23 m6A regulators was evaluated by Spearman correlation analysis in all samples and periodontitis samples only. The expression status differences of 23 m6A regulators between healthy and periodontitis were compared by the Wilcox test. The periodontitis‐related m6A regulators were identified by univariate logistic regression and the cut‐off criteria are *P*‐value <.05. The LASSO (least absolute shrinkage and selection operator) regression was used for feature selection and dimension reduction. Multivariate logistical regression was used to develop m6A regulator related periodontitis classifier. ROC (receiver operating characteristic) curve analysis was used to evaluate the distinguishing performance of the signature.

### 2.3 Correlation analysis between m6A regulators and immune characteristics

Single‐sample gene‐set enrichment analysis (ssgsea) was used to estimate the population of specific infiltrating immunocytes and the activity of specific immune reactions, which define an enrichment score to represent the degree of absolute enrichment of a gene set in each sample within a given data set.^18^ The gene list of infiltrating immunocyte gene‐sets was obtained from the previous study,^17^ and the immune reaction gene‐sets were got from the ImmPort database (http://www.immport.org).^19^ The enrichment scores representing immunocyte abundance and immune reaction activity were compared between healthy and periodontitis samples by the Wilcox test. The correlation of m6A regulators with immunocyte fractions, immune reaction activity and HLA gene expression was determined by Spearman correlation analysis.

### 2.4 Identification of m6A modification pattern

Unsupervised clustering analysis was conducted to identify diverse m6A modification patterns according to the expression of 23 m6A regulators. A consensus clustering algorithm was used to evaluate the cluster numbers and robustness.^15^, ^17^ The R package ‘ConsensuClusterPlus’ implement the above steps for 1000 iterations for guaranteeing the robustness of classification.^20^ PCA was conducted to further validate the 23 m6A regulator expression patterns in different modification patterns. The m6A regulator expression, infiltrating immunocyte abundance score, immune reaction score and HLA gene expression among the 3 distinct modification patterns were compared by the Kruskal test.

### 2.5 Biological enrichment analysis for distinct m6A modification patterns

Biological signalling pathways are very good at reflecting on biological changes; the HALLMARKS pathway and KEGG pathway are two commonly used pathway gene sets. GSVA (gene‐set variation analysis) algorithm was conducted to transform the expression matrix to the pathway activation score matrix, and the pathway activation scores between the two groups were compared by R package ‘limma’.^21^ The gene sets of ‘h.all.v7.0.symbols’ and ‘c2.cp.kegg.v7.0.symbols’ were downloaded from MSigDB database for running GSVA analysis. The differential analysis was set to adjust the *P*‐value <.01 as the cut‐off criterion. The biological function of m6A phenotype‐related genes and m6A regulator‐mediated immune genes was analysed by GO‐BP enrichment analysis using the R package ‘clusterProfiler’.

### 2.6 Identification of m6A mediated genes

To identify m6A regulator meditated genes, samples of 3 distinct m6A modification patterns were analysed by the empirical Bayesian approach of the ‘limma’ R package to identify DEGs between different modification patterns. The criteria for determining significant DEGs were set as the adjusted *P*‐value <.00001. The common m6A regulator‐mediated genes were overlapped by Venn plot. The modification pattern‐related genes and gene modules were identified by WGCNA (weighted gene co‐expression network analysis) using the R package ‘WGCNA’.^22^, ^23^, ^24^

## 3 RESULTS

### 3.1 The landscape of m6A regulators between healthy and periodontitis samples

**FIGURE 1:**
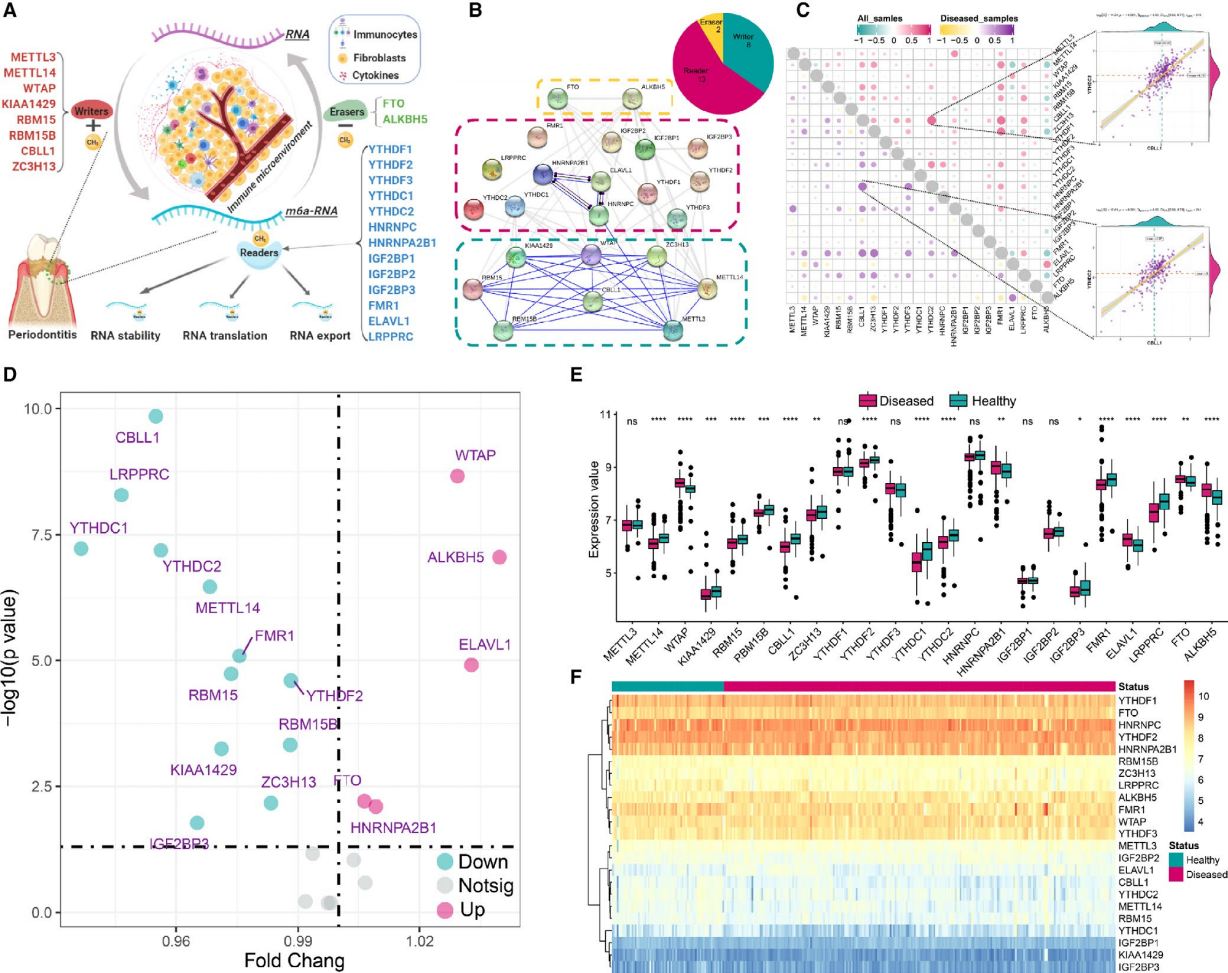
Expression landscape of m6A RNA methylation regulators in periodontitis. (A) The overview of the dynamic reversible process of m6A RNA methylation modification, which regulated by ‘writers’, ‘erasers’ and ‘readers’ in periodontitis and their potential biological functions for RNA. (B) The composition summary of m6A regulators and the protein‐protein interactions among 23 m6A RNA methylation regulators. (C) Correlations among the expression of 23 m6A regulators in all samples and periodontitis samples. The two scatter‐plots demonstrated the most correlated two m6A regulators: YTHDC2 and CBLL1. (D) The volcano plot shows the summary of expression changing information of 23 m6A regulators between healthy and periodontitis samples. (E,F) The box plot and heatmap plot demonstrated the transcriptome expression status of 23 m6A regulators between healthy and periodontitis samples

There are 23 m6A regulators involved in the study, including 8 writers, 13 readers and 2 erasers. The overview of the m6A regulators with their functions in the immune microenvironment was presented (Figure 1A). The regulation interactions of these m6A regulators were exhibited as a protein‐protein network (Figure 1B), and we noticed writers had a very close connection and they usually function as a complex. The transcriptome relationships were investigated, and we found there are close correlations among writers, readers and erasers (Figure 1C); the CBLL1 and YTHDC2 are the most correlated m6A regulators in expression both in all samples and periodontitis samples, indicating that they function together. We also found that FMR1 is highly correlated with many other regulators. Differentially expressed analysis identified 17 expressions altered m6A regulators, where YTHDC1 had the largest fold change and CBLL1 had the most statistically significant change (Figure 1D, Table S1). All erasers had significant expression alterations, while some of the readers did not change significantly, and the well‐studied writer protein METTL3 did not change significantly which suggests that it might not play an important role in periodontitis. (Figure 1E). The 23 m6A regulators were grouped into 4 parts according to their expression level (Figure 1F).

### 3.2 m6A regulators are involved in periodontitis process

**FIGURE 2:**
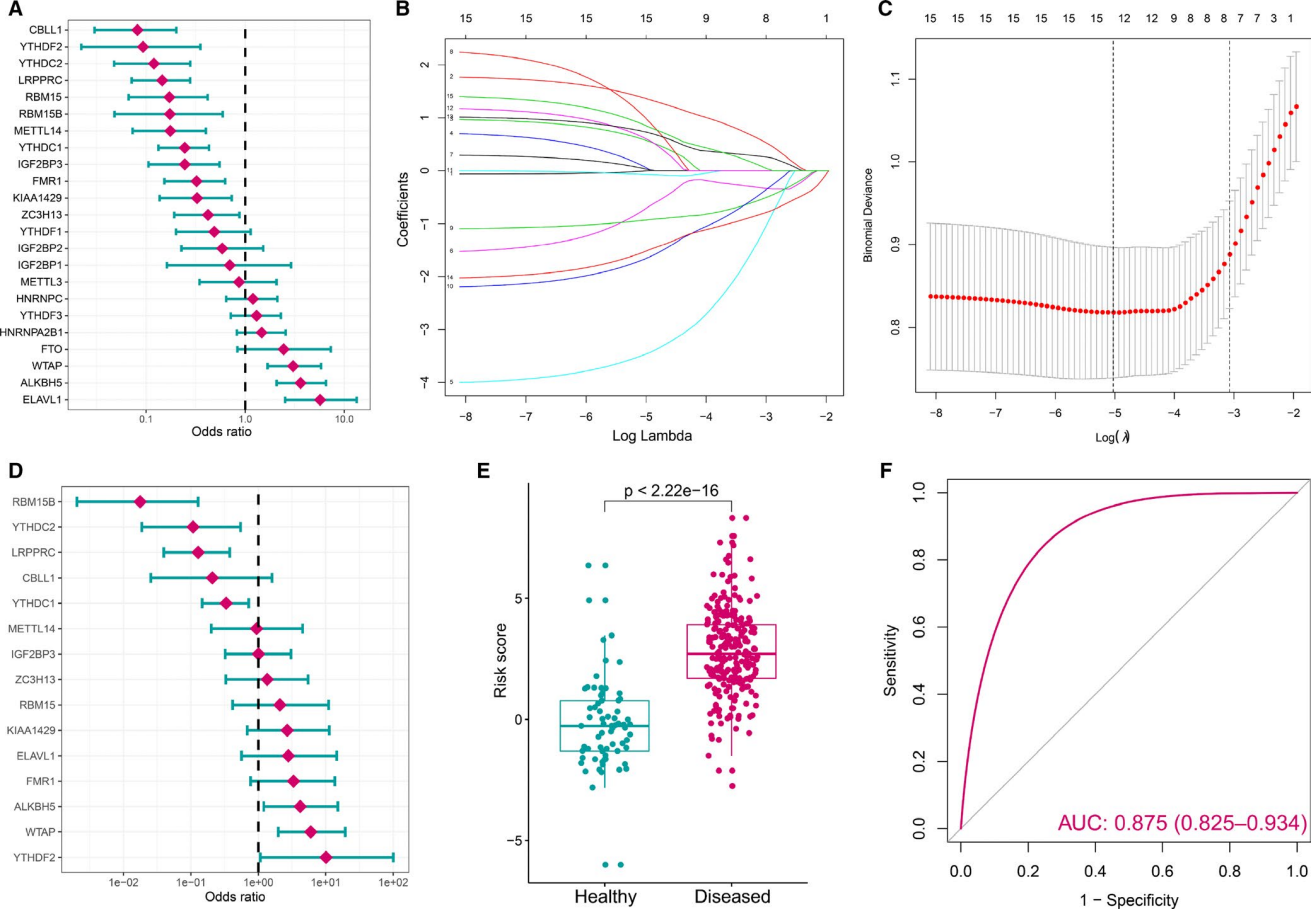
m6A regulators can distinguish healthy and periodontitis samples. (A) Univariate logistic regression investigated the relationship between m6A regulators and periodontitis, revealing 15 periodontitis‐related m6A regulators (*P* < .05). (B) Least absolute shrinkage and selection operator (LASSO) coefficient profiles of 15 periodontitis‐related m6A regulators. (C) 10‐fold cross‐validation for tuning parameter selection in the LASSO regression. The partial likelihood deviance is plotted against log (λ), where λ is the tuning parameter. Partial likelihood deviance values are shown, with error bars representing SE. The dotted vertical lines are drawn at the optimal values by minimum criteria and 1‐SE criteria. (D) Distinguishing signature with 15 m6A RNA methylation regulators was developed by multivariate logistic regression, and the risk scores for periodontitis were calculated. (E) The risk distribution between healthy and periodontitis, where periodontitis have a much higher risk score than healthy samples. (F) Principal component analysis (PCA) of 15 periodontitis‐related m6A regulators between healthy and periodontitis. The two first principal components (PC1, PC2) which explain the most of the variation are plotted. (G) The discrimination ability for healthy and periodontitis samples by m6A regulators was analysed by ROC curve and evaluates by AUC value

To investigate m6A regulators' contribution to the pathogenesis of periodontitis, a series of the bioinformatic algorithm was employed. Univariate logistic regression was used to identify periodontitis‐related m6A regulators, and we found 15 m6A regulators are related to periodontitis (Figure 2A, Table S2). LASSO regression was performed on the 15 periodontitis‐related m6A regulators for feature selection and dimension reduction so that the unimportant regulators could be excluded (Figure 2B,C), and we found all 15 m6A regulators were essential for periodontitis. Multivariate logistic regression was conducted to develop a classifier to distinguish healthy and periodontitis samples (Figure 2D, Table S3). The classifier consisted of 15 m6A regulators that can well distinguish healthy and periodontitis samples according to the risk scores, where periodontitis have a much higher m6A risk score than healthy samples (Figure 2E). And the PCA result also demonstrated a diversity m6A regulator expression pattern that existed between healthy and periodontitis (Figure 2F). ROC curve illustrated the 15 m6A regulators possess a good performance in classifying healthy and periodontitis, indicating m6A regulators indeed play a key role in periodontitis development (Figure 2G).

### 3.3 m6A regulators are associated with immune characteristics of periodontitis

**FIGURE 3:**
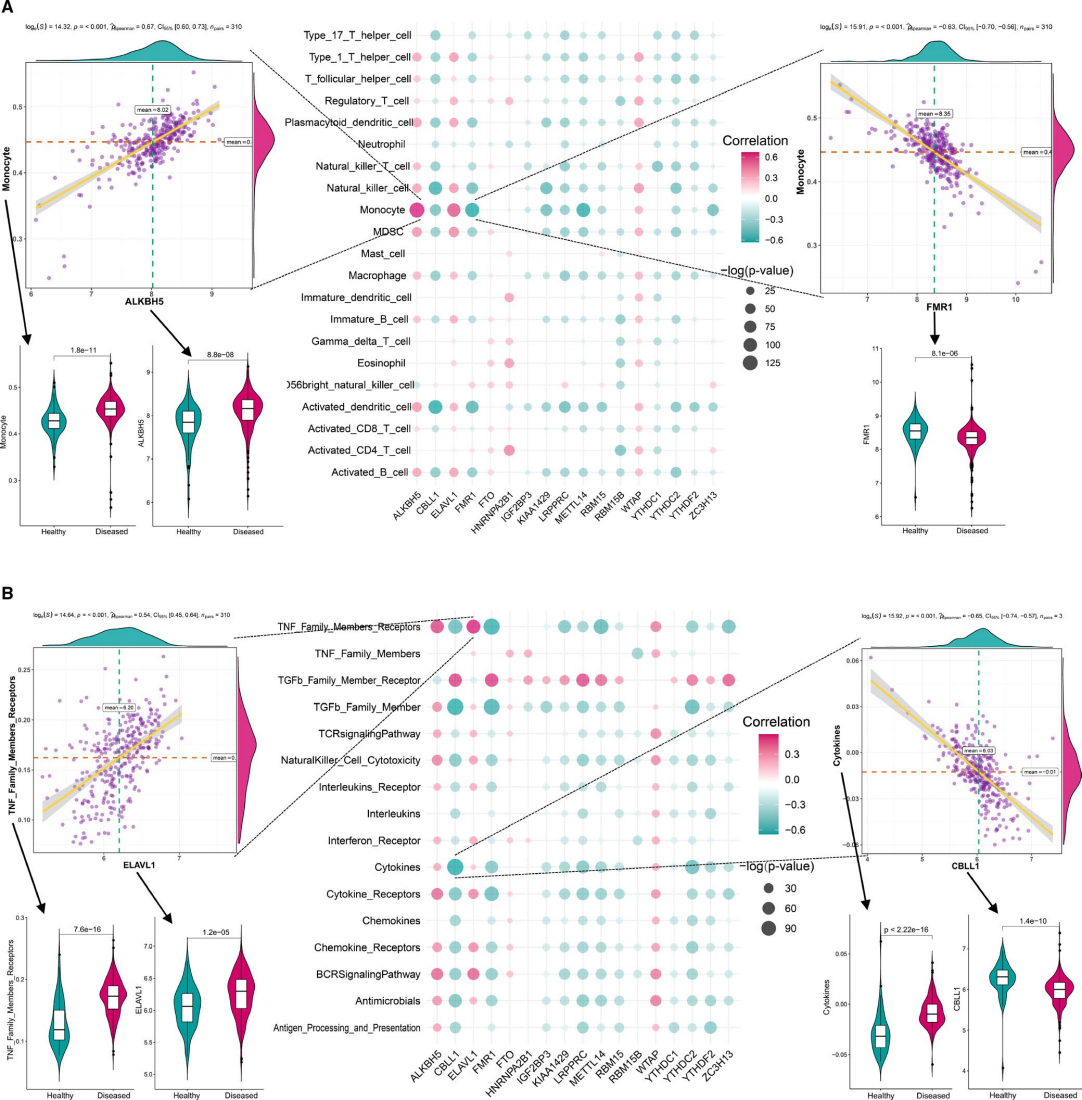
The correlation between infiltrating immunocytes, immune reaction gene‐sets and m6A regulators. (A) The dot‐plot demonstrated the correlations between each dysregulated immune microenvironment infiltration cell type and each dysregulated m6A regulator. The most positive correlated immunocyte‐m6A regulator pair is ALKBH5‐monocyte, and the expression status or fraction status is presented by violin plot at left panel, indicating there are higher expression of ALKBH5 and more monocyte in periodontitis. The most negative correlated immunocyte‐m6A regulator pair is FMR1‐monocyte, and the expression status or fraction status is presented by violin plot at right panel, indicating there are lower expression of FMR1 in periodontitis. (B) The dot plot demonstrated the correlations between each dysregulated immune reaction gene‐set and each dysregulated m6A regulator. The most positive correlated pair is ELAVL1‐TNF_Family_Members_Receptors, and the expression status or activity status is presented by violin plot at left panel, indicating there are higher expression of ELAVL1 and more active TNF_Family_Members_Receptors reaction in periodontitis. The most negative correlated pair is CBLL1‐cytokine, and the expression status or activity status is presented by violin plot at right panel, indicating higher CBLL1 expression and less active cytokine reaction in periodontitis

To investigate the biological behaviours among m6A regulators and immune microenvironment, we performed correlation analysis for dysregulated m6A regulators with infiltrating immunocytes, immune reaction gene‐sets and HLA gene expression as our previous study did and obtained the same results in some aspects.^25^ The difference in the abundance of 23 immune microenvironment infiltrating cells between healthy and periodontitis samples was revealed (Figure S1A, Tables S4 and S5). Most infiltrating immunocyte fractions changed in periodontitis such as activated B cells including innate immunity and adaptive immunity. Correlation analysis found m6A regulators are closely related to many immunocytes (Figure 3A). For instance, monocyte abundance is positively correlated with ALKBH5 and negatively correlated with FMR1, indicating increased monocytes infiltrated in periodontitis which is closely affected by the expression of ALKBH5 and FMR1. Similarly, we analysed immune reactions and HLA in periodontitis. The difference in the activity of each immune reaction gene‐set between healthy and periodontitis samples was presented (Figure S1B, Tables S6 and S7). A lot of immune reactions have increased or decreased in periodontitis such as the higher activity of cytokine and cytokine receptor reactions occurred in periodontitis. We found ELAVL1 and CBLL1 have the most correlated and significant immune gene‐sets, where ELAVL1 is positively correlated with TNF_Family_Members_Receptors, and CBLL1 is negatively correlated with cytokine activity (Figure 3B). This indicated ELAVL1 andCBLL1 played an important role in TNF or cytokine reactions in periodontitis. The expression status of HLA was explored and most of which were changed (Figure S2A, Tables S8 and S9). ALKBH5‐HLA_B is the most positively correlated pair, and the most negatively correlated HLA‐m6A pair is LRPPRC‐HLA_DOA (Figure S2B).

### 3.4 m6A RNA methylation modification patterns mediated by 23 regulators in periodontitis

**FIGURE 4:**
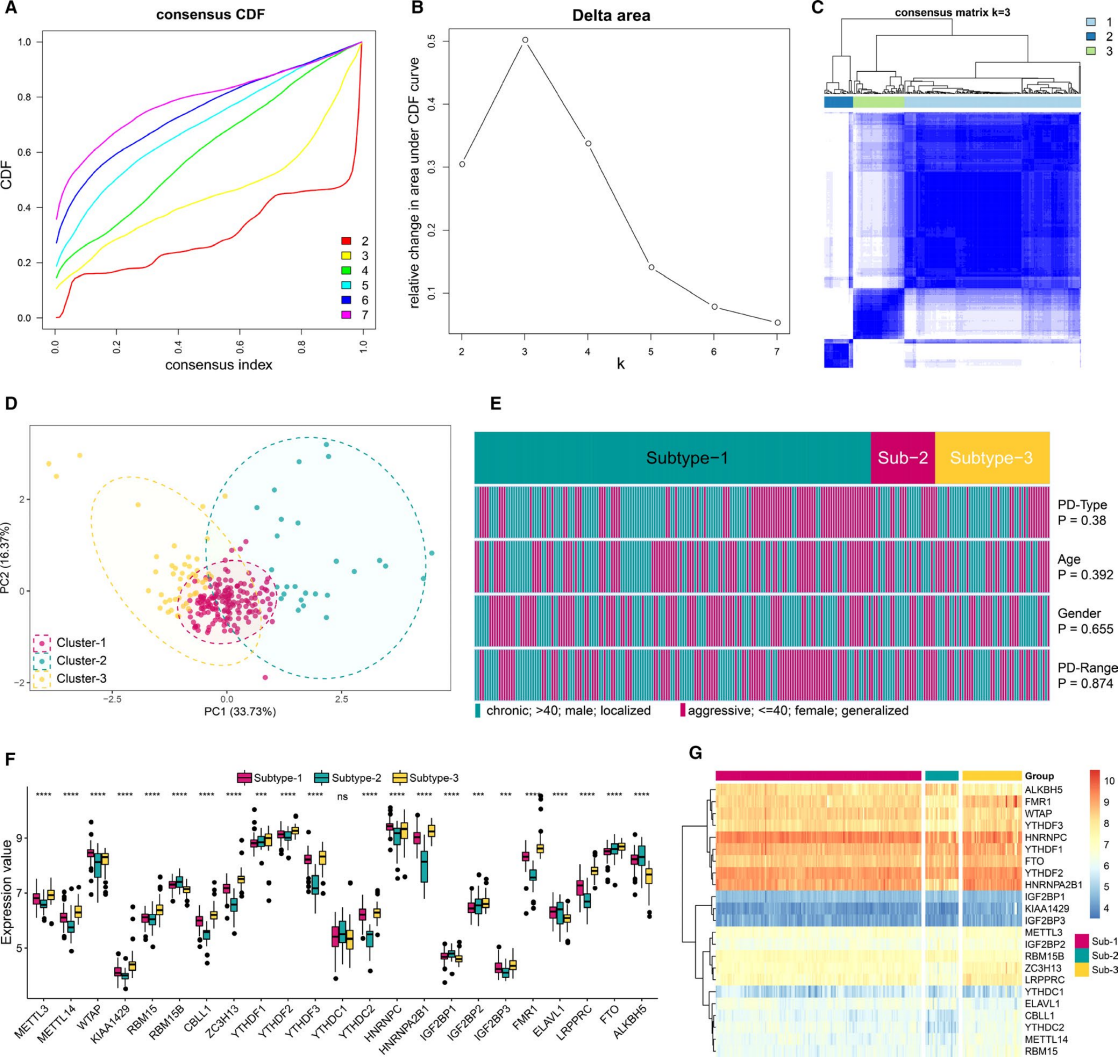
Unsupervised clustering of 23 m6A regulators. Identifying 3 distinct m6A modification pattern subtypes in periodontitis. (A) Consensus clustering cumulative distribution function (CDF) for k = 2‐7. (B) Relative change in area under CDF curve for k = 2‐7. (C) Heatmap of the matrix of co‐occurrence proportions for periodontitis samples. (D) Principal component analysis for the transcriptome profiles of 3 m6A subtypes, showing a remarkable difference on transcriptome between different modification patterns. (E) Comparing of age, gender, periodontitis range and periodontitis type among 3 m6A modification patterns. The heatmap illustrates the association of different clinical characters with the 3 subtypes. (F) The expression status of 23 m6A regulators in the three m6A subtypes. (G) Unsupervised clustering of 23 m6A regulators in the 3 modification patterns

To investigate m6A modification patterns in periodontitis, we conducted unsupervised consensus clustering analysis for periodontitis samples based on the expression of 23 m6A regulators (Figure 4A‐C, Table S10). Three distinct subtypes of periodontitis were identified with qualitatively different expression of 23 m6A regulators, including 166 samples in subtype‐1, 27 samples in subtype‐2 and 48 samples in subtype‐3 (Figure 4D). The 3 distinct modification pattern grouping is different from the current periodontitis classification, and there are no obvious differences of clinical characteristics among different modification patterns (Figure 4E). All 23 m6A regulators have remarkable differences in expression between 3 m6A modification patterns except for YTHDC1 (Figure 4F,G), validating the existence of diversity m6A modification patterns in periodontitis.

### 3.5 Immune microenvironment characteristics in distinct m6A modification patterns

**FIGURE 5:**
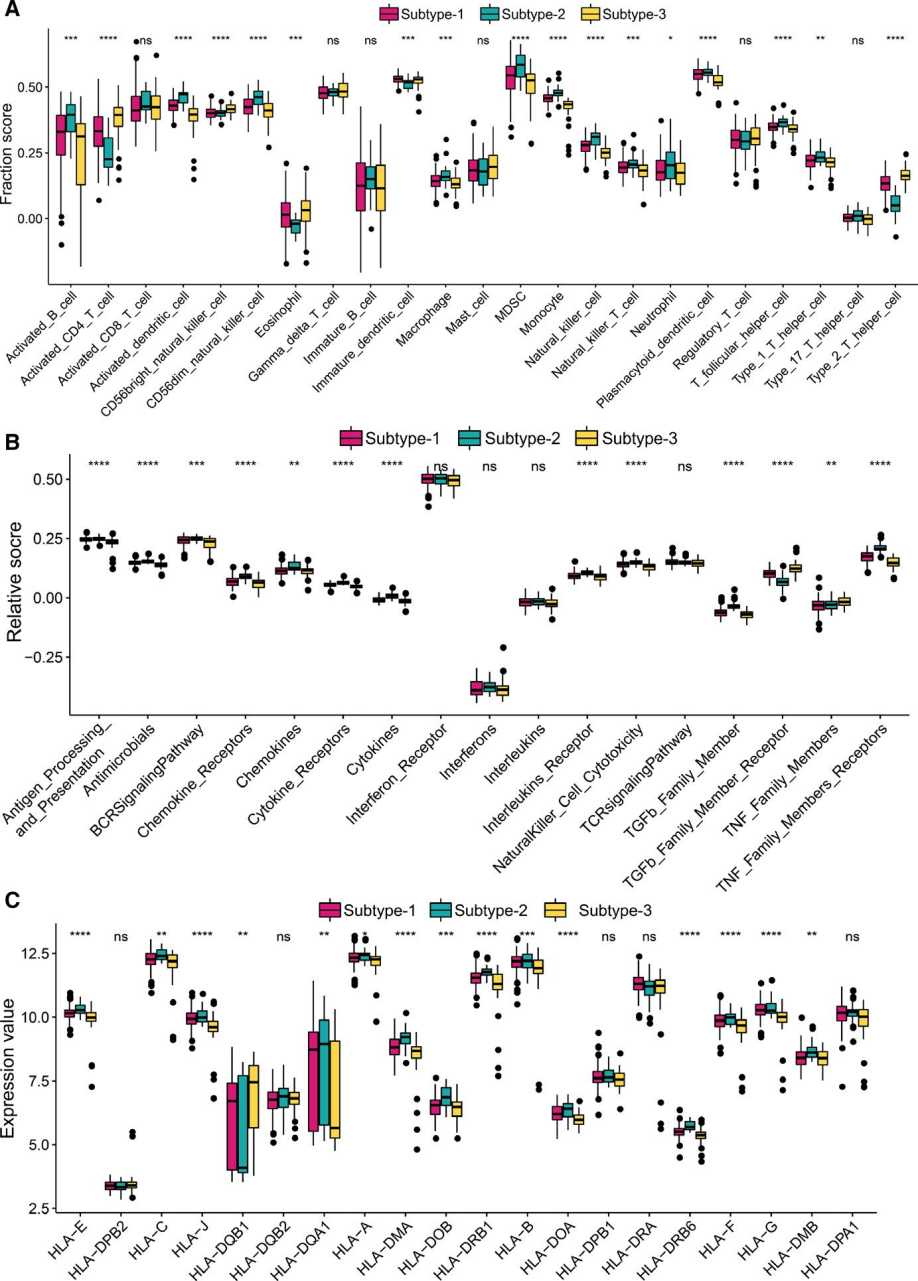
Diversity of immune microenvironment characteristics among distinct m6A modification patterns. (A) The abundance differences of each immune microenvironment infiltrating immunocyte in 3 m6A modification patterns. (B) The activity differences of each immune reaction gene‐set in 3 m6A modification patterns. (C) The expression differences of each HLA gene in 3 m6A modification patterns

To find out the differences of immune microenvironment characteristics among these distinct m6A modification patterns, infiltrating immunocytes, immune reaction gene‐sets and HLA gene expression were evaluated. We found many immunocytes are differing among 3 patterns (Figure 5A). Pattern‐1 has a relatively low infiltrated immunocytes compared with pattern‐2 and pattern‐3. Pattern‐2 has a higher level of infiltrated activated B cell, activated dendritic cell, CD56dim natural killer cell, MDSC, natural killer T cell, neutrophil, T follicular helper cell, type‐1 T helper cell and type‐17 T helper cell, while activated CD4 T cell, mast cell and type‐2 T helper cells are enriched in pattern‐3. As for immune reactions, pattern‐2 has medium immune reactions, while pattern‐2 and pattern‐3 have more active immune reactions, especially pattern‐2. For example, chemokines and chemokine receptors' immune reactions are very active in pattern‐2 and TGFb family member receptors in pattern‐3 (Figure 5B). Similar trends are observed in HLA gene expression (Figure 5C). These results suggested pattern‐2 and pattern‐3 m6A modification mediate an active immune response, and pattern‐1 leads to a mild immune response in periodontitis, while the immune response mediated by pattern‐2 and pattern‐3 is different. The above results once again proved that m6A methylation modification had an essential regulatory role in shaping different immune microenvironments in periodontitis.

### 3.6 Biological characteristics of 3 m6A modification patterns

**FIGURE 6:**
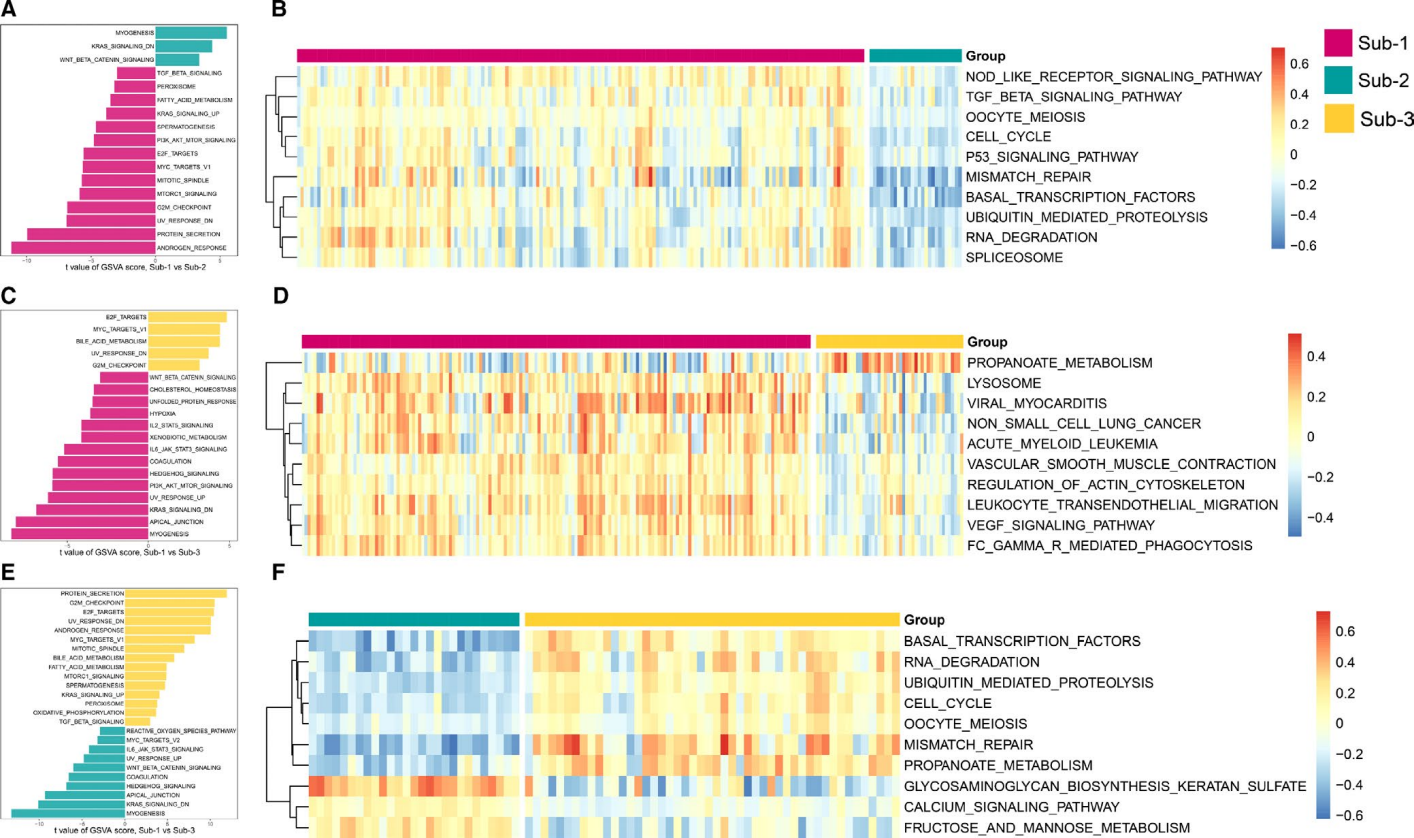
The underlying biological function characteristics diversity among 3 m6A modification patterns. (A,B) The differences of HALLMARKS pathway and KEGG pathway enrichment score between m6A modification pattern 1 and pattern 2 (A for HALLMARKS pathway and B for KEGG pathway). (B) The differences of HALLMARKS pathway and KEGG pathway enrichment score between m6A modification pattern 1 and pattern 3 (C for HALLMARKS pathway and D for KEGG pathway). (C) The differences of HALLMARKS pathway and KEGG pathway enrichment score between m6A modification pattern 2 and pattern 3 (E for HALLMARKS pathway and F for KEGG pathway)

**FIGURE 7:**
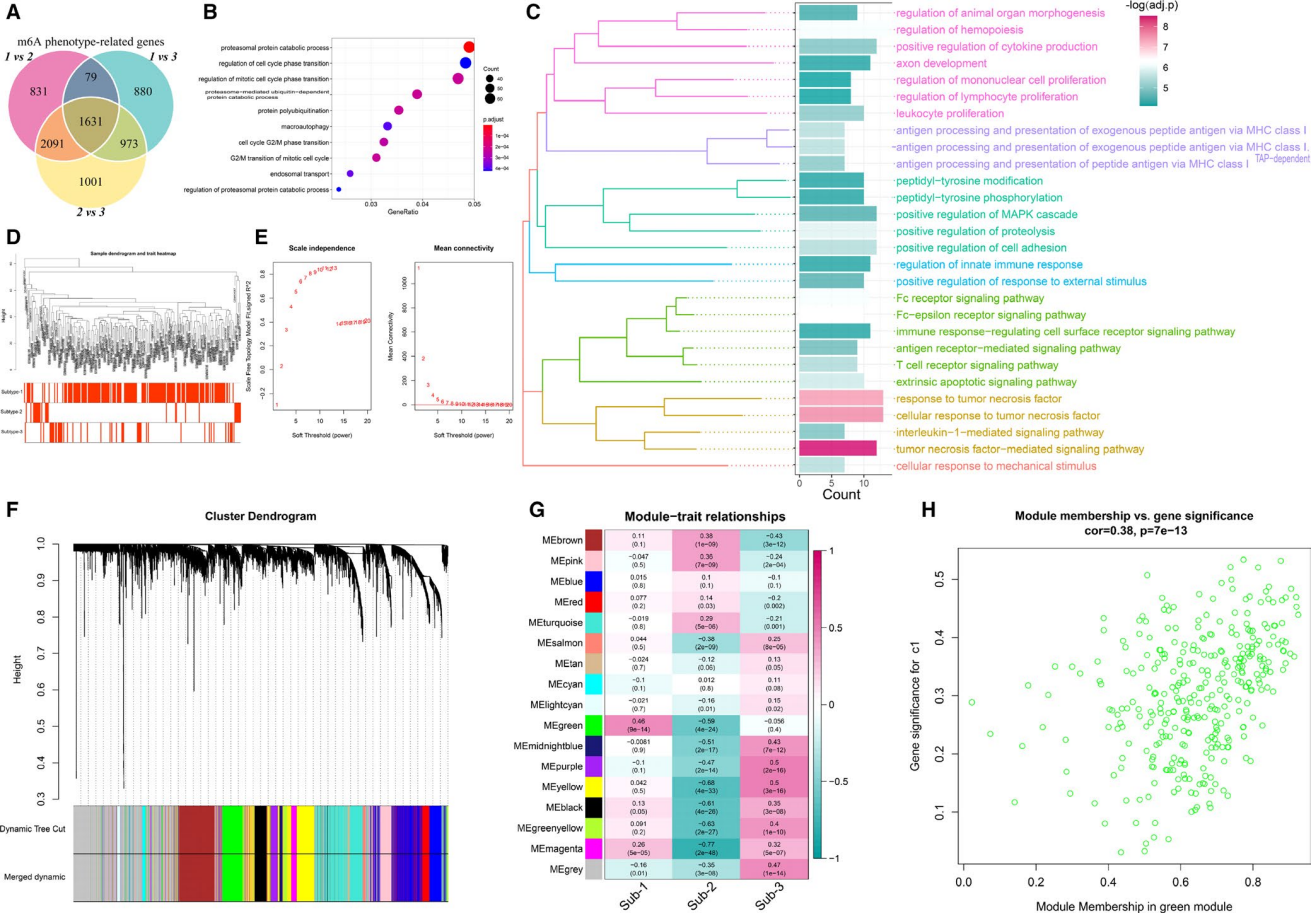
Identification and function analysis of m6A phenotype‐related genes in periodontitis. (A) 1631 m6A phenotype‐related genes shown in Venn diagram. (B) GO‐BP functional enrichment analysis revealed the biological characteristics of m6A phenotype‐related genes. (C) The GO‐BP enrichment analysis for the m6A phenotype‐related immune genes uncover the relationship between m6A regulators and immune regulations. GO categories are grouped according to functional. (D) The sample clustering was based on the expression data of all samples. The top 25% variation genes was used for the analysis by WGCNA, and outlier samples were excluded. (E) Analysis of the scale‐free ft index and analysis of the mean connectivity for various soft‐thresholding powers. (F) Gene dendrogram obtained by average linkage hierarchical clustering. The colour row underneath the dendrogram shows the module assignment determined by the Dynamic Tree Cut, in which 16 modules were identified. (G) Heatmap of the correlation between module eigengenes and the m6A modification patterns. (H) A scatterplot of gene significance (GS) for m6A modification pattern‐1 vs module membership (MM) in the green module. GS and MM exhibit a very significant correlation, implying that hub genes of the green module also tend to be highly correlated with m6A modification pattern‐1

To investigate the biological reactions in the 3 m6A modification patterns, we compared the HALLMARKS pathway and KEGG pathway between each of them, and GSVA enrichment analysis was applied to evaluate the activation status of biological pathways. Compared with pattern‐2 and pattern‐3, pattern‐1 has more enriched pathways such as the famous PI3K‐AKT pathway (Figure 6A‐D). Pattern‐2 and pattern‐3 had nearly the same amount of enriched pathways (Figure 6E,F). To further understand the molecular mechanisms by which genes are involved in m6A regulator mediated regulations, we identified the m6A phenotype‐related DEGs and overlapped them to get the m6A phenotype‐related genes. A total of 1631 common genes were regarded as m6A phenotype‐related genes, and GO enrichment analysis revealed that they are mainly involved in the regulation of cell cycle and protein modification (Figure 7A,B, Table S11). The m6A phenotype‐related genes which take part in immune regulation were screened out and of the enriched biological processes were remarkably related to the antigen process, Fc signalling transduction, regulation of immunocyte proliferation and cytokine‐mediated pathways (Figure 7C). A comprehensive gene landscape correlated with each m6A modification pattern has been constructed, and gene‐gene modules related to different m6A modifications were identified by WGCNA as our previous study did (Figure 7D‐F, Table S12)^25^. Sixteen gene modules were determined, and different modification pattern matched their related genes (Figure 7G), such as m6A regulator modification pattern‐1 closely related to genes in green modules (Figure 7H). These results can shed light on gene expression regulation network mediated by m6A regulators.

## 4 DISCUSSION

Periodontitis is a chronic inflammatory disease that involves complex interactions between pathogens and immune reactions.^1^ Accumulating evidence confirmed the indispensable role of m6A modification in both innate and adaptive immune reactions.^26^ So far, studies have been carried out to explore m6A in immunity, especially in tumour microenvironment infiltrating cells, and the results confirmed its fundamental role in tumour immunity.^11^, ^27^, ^28^ Thus, we believe that similar results concerning m6A modification in shaping the immune microenvironment in periodontitis could be observed. To well illustrate these questions, here, we systematically investigated the m6A modification patterns in the immune microenvironment of periodontitis. To unravel how m6A could shape the immune reactions in periodontitis, activate immune pathways as well as enrich infiltrating immunocytes, a series of analysis was performed and we made the following findings. First, we found the expression of most m6A regulators altered between healthy and periodontitis, suggesting m6A regulators are involved in periodontitis development. A classifier established by m6A regulators can well distinguish healthy and periodontitis samples, confirming the important role of m6A regulators in periodontitis again. Among 23 m6A regulators, YTHDC1, WTAP and ALKBH5 may be the most important ones because of their large fold change and significance in multivariate analysis. Besides, many of m6A regulator have protein interaction or expression correlation, revealing the regulating network of m6A modification. Second, the correlations between m6A regulators and immune characteristics of periodontitis were explored, including infiltrating immunocytes, immune reaction gene‐sets and HLA gene expression. We found many m6A regulators are closely related to these immune characteristics, implying the essential role of m6A modification in periodontitis immune microenvironment regulation. Infiltrating monocyte abundance is positively associated with ALKBH5 and negatively with FMR1. Monocytes are an important component of innate immunity and play a significant role in periodontitis homeostasis.^29^ The previous study demonstrated FMR1 CGG repeated expansions are associated with cytokines of monocyte, while there are no related reports for ALKBH5 and monocyte.^30^ CBLL1 is related to several immune reactions, and especially cytokine has been reported taking part in periodontitis progress and has essential and pleiotropic impacts on the recruitment of specific immunocytes, control of pathobionts and induction or suppression of osteoclastic activity.^31^ There are no related researches about CBLL1 in cytokine signalling and immune regulations, but it has been reported METTL3 depletion enhanced proinflammatory cytokine expression in osteoblast and METTL3 promotes cytokine inflammatory response of osteoarthritis.^32^, ^33^ These findings may point out the direction of the m6A immune regulation mechanism in periodontitis. Third, unsupervised clustering of the periodontitis samples using m6A regulators expression profiles led us to three subtypes with distinctive m6A modification pattern, and each subtype has its unique immune characteristics. The modification of pattern‐2 and pattern‐3 has more infiltrating immunocytes and more active immune reactions compared with pattern‐1. Considering the immune characteristics of each subtype, it confirmed the reliability of our classification of immune phenotypes for different m6A regulator. This classification strategy for immune subtype can help us understand the underlying mechanism of immune regulation so that precise therapeutic methods can be applied and periodontitis can be subtyped from the molecular level or immune level not only the phenotype level^25^. A recent study used this method to identify 3 distinct m6A modification patterns in gastric cancer, and the results enhanced the understanding of tumour microenvironment, which can help us for more effective immunotherapy strategies.^17^ Molecular subtyping strategy is widely used in the oncology field, and identification of novel molecular subtypes can make a better treatment plan.^34^, ^35^ For periodontitis, Kebschull et al^13^ used dysregulated genes in periodontitis to identify two subtypes of periodontitis which is different from the currently accepted periodontitis classification, and the two novel subtypes manifest significant diversity in extent and severity of periodontitis, the intensity of serum antibody responses to periodontal microbiota and level of subgingival colonization. Therefore, the 3 distinct m6A modification pattern subtypes in periodontitis demonstrated that m6A modification pattern of gingival tissues can indeed be regarded as an alternative pathobiology‐based classification of periodontitis, which is related to phenotypic features of the disease. Last, the m6A regulator‐related genes and m6A modification pattern genes were identified. These genes' expression regulation was influenced by m6A modification, and revealing the biological function of them can contribute to the illustration of periodontitis pathogenesis from the m6A modification view. Subtype‐2 have more activation in famous signalling pathway of IL6‐STAT3, and at the same time more activated B cell infiltrating are seen in subtype‐2. ALKBH5 is also up‐regulated in subtype‐2 and periodontitis; these results may suggest ALKBH5, IL6‐STAT3, activated B cells and periodontitis have great correlations. The abundance results in our study can give a lot of these similar correlations and other researchers in the field will get directions to catch the key m6A regulator and immune features in periodontitis rapidly. That is one of the most important scientific meaning for our study.

Our study is the first one to systematically analyse the relationship between m6A regulator modification and immune microenvironment. Abundance results were generated, and they can lead to a new direction for immune‐related periodontitis pathogenesis researches from the m6A modification mechanism. We are the first one to introduce the latest m6A mechanism in periodontitis, and m6A modification was confirmed to be involved in the regulation of the immune microenvironment in periodontitis. Epigenetic related research in the field of periodontitis is inherently a handful; the gap is very big. Combining the latest m6A mechanism and immune microenvironment theory to reveal periodontitis pathogenic mechanisms, such research in periodontitis is groundbreaking, to a large extent can supplement the gap of periodontitis in epigenetic modifications or RNA modified. This study will inspire many researchers to carry out m6A‐related studies in the field of periodontitis, and the numerous results of this study can offer them a better direction. However, this study also has some shortcomings that we must admit. First of all, this study is based on bioinformatics analysis, and many results are theoretically valid, not verified by experiments, the accuracy of which remains to be verified. But from the results of numerous tumour studies based on TCGA data analysis, we have confidence that the results of bioinformatics analysis are reliable. Nonetheless, the experiment is the only criterion for testing, and our research results need to be verified by subsequent experiments. The analysis of immune cells also used the most widely applied analytical method to quantify the amount of immunocytes, but to get the most accurate amount of immunocytes still requires single‐cell sequencing. Besides, some data were unavailable to us, such as microbial plaque information, serum detection results and clinical characteristics of periodontitis, so it is difficult to reveal the important role of m6A in immune regulation from multiple dimensions to a large extent, and the analysis results are relatively single. We hope to obtain this part of the data later to reveal more useful results. Besides, another independent data set (GSE10334 which contains 64 healthy and 183 periodontitis samples) was involved in external validation and the aberrant expression of 23 m6A regulators between healthy and periodontitis samples was validated by it. External data set validation could be a substitution for experiment validation at some point. Similar expression patterns were found between healthy and periodontitis samples in this data set which proved our findings to be solid (Figure S3). This strategy are widely used in this type of studies and some recently high‐quality studies performed this analysis.^36^, ^37^, ^38^ Nevertheless, all of our findings did confirm the strong impact m6A modification has on the immune characteristics of periodontitis and have provided new insights into understanding the pathogenesis of periodontitis.

## 5 CONCLUSIONS

In conclusion, our study revealed the underlying regulation mechanisms of m6A methylation modification in the periodontitis immune microenvironment. The diversity of m6A modification patterns has a non‐negligible impact on the heterogeneity and complexity of the immune microenvironment. The comprehensive analysis of the periodontitis m6A modification pattern will make a great contribution to understanding the underlying mechanism of the immune regulation network in periodontitis, inspiring more effective therapeutic methods.

## CONFLICT OF INTEREST

The authors confirm that there are no conflicts of interest.

## AUTHOR CONTRIBUTIONS

**Xiaoqi Zhang:** Formal analysis (lead); methodology (lead); writing‐original draft (lead). **Shizhen Zhang:** Data curation (lead). **Xinyu Yan:** Software (lead); visualization (lead). **Yue Shan:** Methodology (lead). **Lu Liu:** Validation (equal). **Jing Zhou:** Validation (equal). **Qianyun Kuang:** Visualization (supporting). **Minqi Li:** Supervision (equal); writing‐review & editing (equal). **Hu Long:** Project administration (equal). **Wenli Lai:** Conceptualization (equal); data curation (equal); funding acquisition (lead); project administration (equal); supervision (equal); validation (equal); writing‐review & editing (equal).

## Supporting information

Fig S1‐S3

Table S1‐S12

## Data Availability

The data that support the findings of this study are available in [https://www.ncbi.nlm.nih.gov/geo/query/acc.cgi?acc=gse16134].
