# Supplementary material for: m6A regulator‐mediated RNA methylation modification patterns are involved in immune microenvironment regulation of periodontitis
Source: J Cell Mol Med. 2021 Mar 16;25(7):3634–45. doi: 10.1111/jcmm.16469 (PMC8034465; doi:10.1111/jcmm.16469)
Supplement: Supplementary file 1 — Fig S1‐S3 [file JCMM-25-3634-s001.docx]

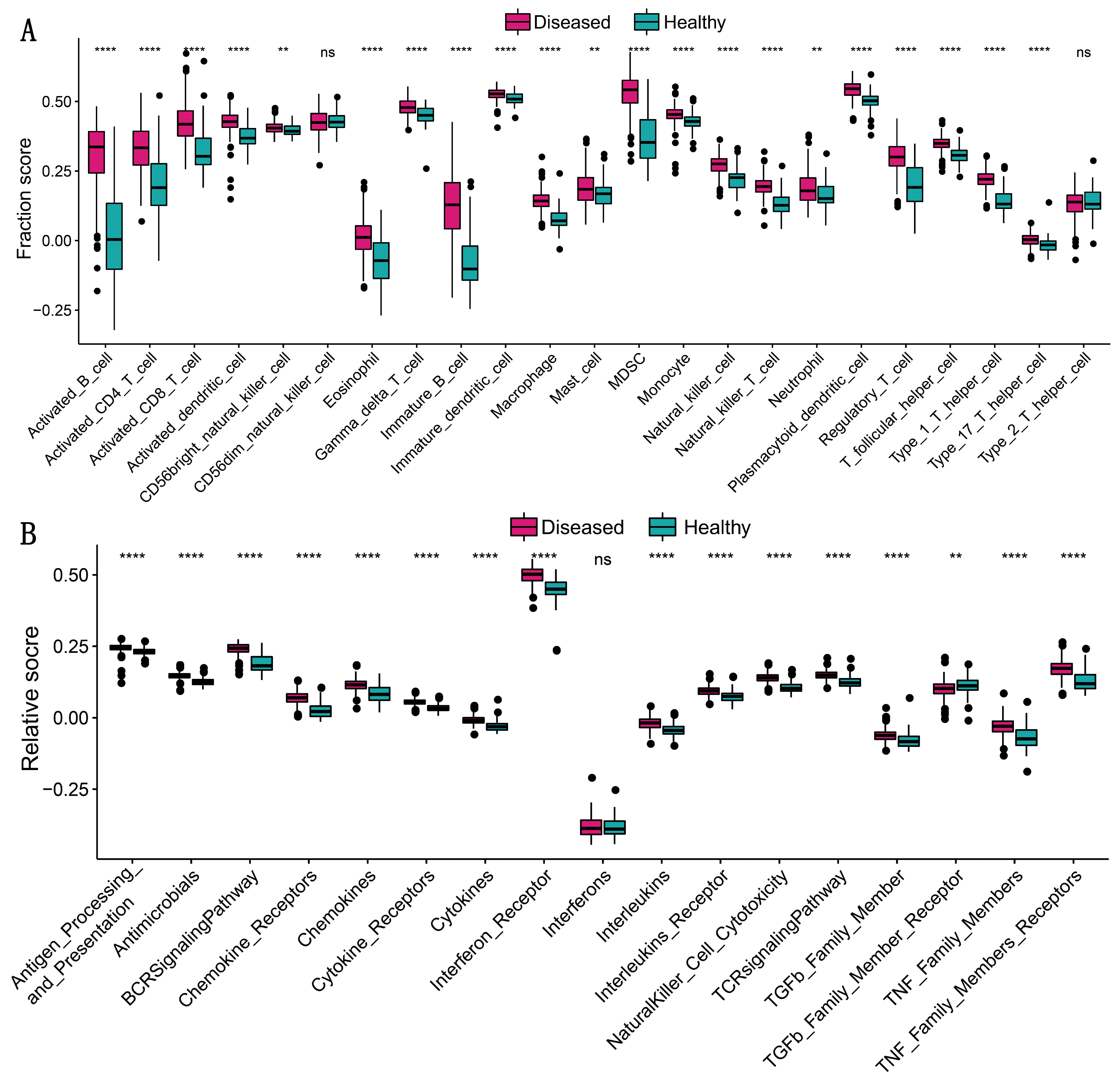


Figure S1. (A) Difference in the abundance of each immune microenvironment infiltrating cell between healthy and periodontitis samples. (B) Difference in the activity of each immune reaction gene-set between healthy and periodontitis samples.


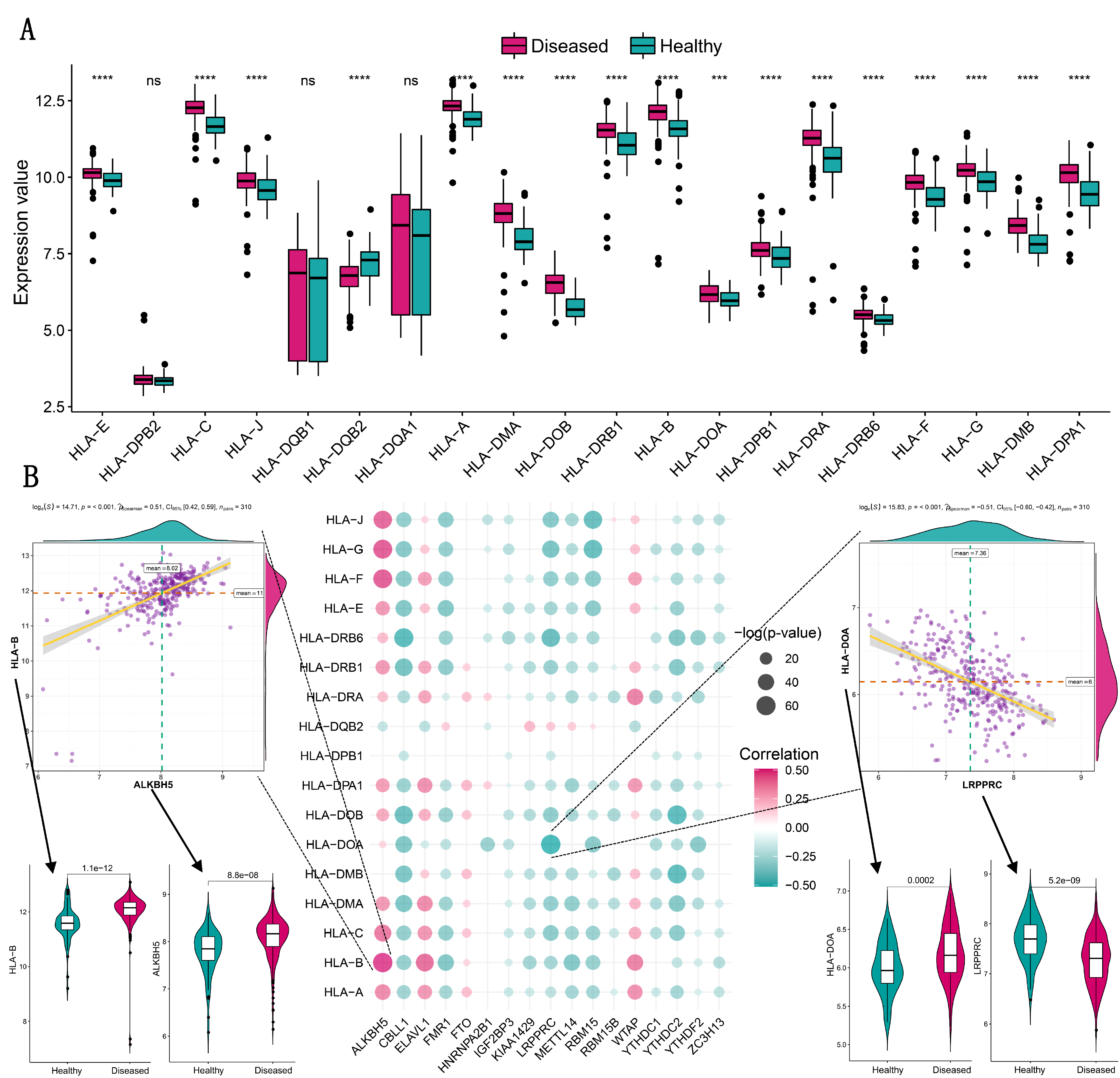


Figure S2. The correlation between HLA and m6A regulators. (A) Difference in the transcriptome expression of each HLA gene between healthy and periodontitis samples. (B) The dot-plot demonstrated the correlations between each dysregulated HLA gene and each dysregulated m6A regulator. The most positive correlated HLA-m6A pair is ALKBH5-HLA_B and the expression are presented by violin-plot at left panel, indicating there are higher expression of ALKBH5 and HLA-B in periodontitis. The most negative correlated HLA-m6A pair is LRPPRC-HLA_DOA and the expression status are presented by violin-plot at right panel, indicating low expression of LRPPRC and high expression of HLA-DOA in periodontitis.


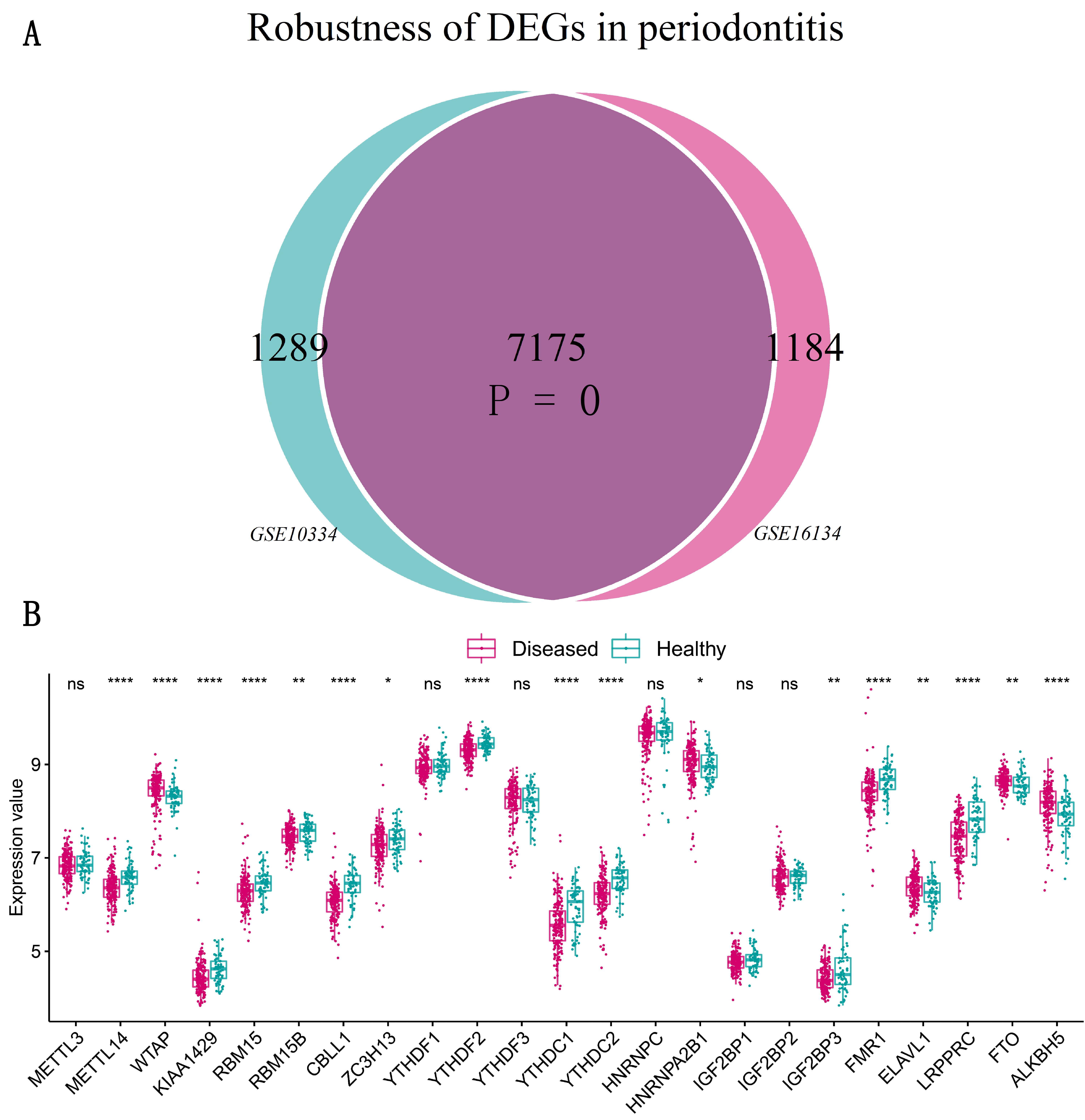


Figure S3. External validation for m6A regulators. (A) The overlapping of DEGs between GSE16134 and external validation-set GSE10334. The hypergeometric test revealed the DEGs in two datasets are highly in according, indicating the robustness of DEGs in periodontitis. (B) The expression patterns of 23 m6A regulators in external validation. It can be seen the 23 m6A regulator expression patterns were same as our study and this could be a substitution for qPCR validation.
